# Structural changes in the temporal lobe and piriform cortex in frontal lobe epilepsy

**DOI:** 10.1016/j.eplepsyres.2014.03.001

**Published:** 2014-07

**Authors:** M. Centeno, C. Vollmar, J. Stretton, M.R. Symms, P.J. Thompson, M.P. Richardson, J. O’Muircheartaigh, J.S. Duncan, M.J. Koepp

**Affiliations:** a Department of Clinical and Experimental Epilepsy, UCL Institute of Neurology, Queen Square, NSE MRI Unit, National Society for Epilepsy, Chalfont St Peter SL9 0RJ, UK; b Neurology Department, Hospital Universitario Vall d’Hebron, Universitat Autonoma de Barcelona, Paseo Vall d’Hebron, 119, 08035 Barcelona, Spain; c Epilepsy Centre, Department of Neurology, University of Munich, Germany; d Department of Clinical Neuroscience, Institute of Psychiatry, King's College, De Crespigny Park, London SE5 8AF, UK

**Keywords:** Frontal lobe epilepsy, Piriform cortex, Voxel based morphometry, MRI

## Abstract

**Background:**

Neuronal networks involved in seizure generation, maintenance and spread of epileptic activity comprise cortico-subcortical circuits. Although epileptic foci vary in location across focal epilepsy syndromes, there is evidence for common structures in the epileptogenic networks. We recently reported evidence from functional neuroimaging for a unique area in the piriform cortex, common to focal epilepsies in humans, which might play a role in modulating seizure activity.

In this study, we aimed to identify common areas of structural abnormalities in patients with frontal lobe epilepsy (FLE).

**Methods:**

T1-weighted MRI scans of 43 FLE patients and 25 healthy controls were analysed using voxel based morphometry. Differences in regional grey matter volume were examined across the whole brain, and correlated with age at epilepsy onset, duration and frequency of seizures.

**Results:**

We detected areas of increased grey matter volume in the piriform cortex, amygdala and parahippocampal gyrus bilaterally, as well as left mid temporal gyrus of patients relative to controls, which did not correlate with any of the clinical variables tested. No common areas of atrophy were detected across the FLE group.

**Conclusions:**

Structural abnormalities within the piriform cortex and adjacent structures of patients with FLE provide further evidence for the involvement of this area in the epileptogenic network of focal epilepsies. Lack of correlation with duration or age of onset of epilepsy suggests that this area of abnormality is not a consequence of seizure activity.

## Highlights

• Voxelwise analysis of structural images is performed in a group of patients with FLE.
• Structural abnormalities are found in piriform cortex and adjacent structures including amygdala.
• These common areas of structural abnormality are seen independently of the side of seizure focus lateralisation.
• This is concordant with previous findings in functional imaging analysis of patients with focal epilepsy.

## Introduction

Changes in grey matter volumes (GMV) have been reported in a number of epilepsy syndromes (Bernasconi et al., 2004; Keller et al., 2002; Lawson et al., 2002; Widjaja et al., 2011; Woermann et al., 1999). Regional increases and decreases of GMV have been identified within the epileptogenic region but also extending to brain areas distant from the seizure onset zone. Atrophy secondary to neuronal loss is the common pathological correlate of decreased GMV in the epileptogenic zone (Bernasconi et al., 2004; Keller et al., 2002). However, the biological significance of changes remote from the epileptic focus remains unclear.

In focal epilepsies, the network involved in the generation, modulation and spread of seizures may encompass not only the seizure onset zone but a number of areas believed to be involved in seizure modulation (Norden and Blumenfeld, 2002). Although seizure onset zones vary across different focal epilepsies, there is evidence for common cortico-subcortical circuits that underlie the maintenance and propagation of seizures. Animal and human studies have shown that areas comprising the nigro-striatial pathways, thalamus (Norden and Blumenfeld, 2002) are key parts of the epileptogenic network in both focal and generalised epilepsies. We recently reported evidence from functional neuroimaging for a unique area in the piriform cortex, common to focal epilepsies in humans, which might play a role in modulating seizure activity (Laufs et al., 2011).

Structural changes in patients with TLE have been widely studied using region and voxel-based morphometry (VBM) analysis (Bernasconi et al., 2004; Keller et al., 2002); however, these studies are usually dominated by areas of atrophy in the hippocampus and ipsilateral temporal lobe, which affects the accuracy of the normalisation process involved in this type of analysis. Only few studies have assessed structural abnormalities in patients with frontal lobe epilepsies (FLE) (Lawson et al., 2002; Widjaja et al., 2011). In this study we used whole brain VBM analysis of grey matter to explore common structural changes in a population with FLE.

## Materials and methods

We recruited 43 patients with drug resistant FLE (26 left FLE and 17 right FLE). Diagnosis and lateralisation of seizure focus was performed by experienced neurologists based on video-EEG, seizure semiology, MRI imaging and FDG-PET/Ictal SPECT when available. The aetiology was cryptogenic in 32 patients. Small areas of focal cortical dysplasia in concordance with the suspected seizure onset zone were identified in 11 patients. Additionally, we scanned 25 healthy controls with no history of neurological or psychiatric disorders. Population characteristics are reported in Table 1.

The study was approved by the Research Ethics Committee of the UCL Institute of Neurology and UCL Hospitals.

Subjects were scanned with a 3 T General Electric Excite HD scanner. A 3-dimensional T1-weighted fast spoiled gradient echo (FSPGR) volumetric scan was obtained for each subject. Matrix size was 256 × 256 × 196 voxels, with an isotropic voxel size of 1.1 mm (echo time/repetition time/inversion time 2.8/6.6/450 ms, flip angle 20°).

T1 images were processed and analysed using Statistical Parametric Mapping software (SPM8) (http://www.fil.ion.ucl.ac.uk/spm8).

Segmentation of the T1 images was performed using the “New segmentation” algorithm of SPM8. The grey matter, white matter and CSF tissue maps where normalised to MNI space using the *DARTEL* toolbox. The resulting tissue classification GM images were modulated by the Jacobian determinants derived from the registration step, in order to preserve subject's tissue volume after warping. Finally, images were smoothed by an 8-mm full width at half maximum isotropic Gaussian kernel.

Voxel-wise GMV differences between FLE patients and controls were examined using independent-sample *t*-tests. To account for differences in brain sizes, images were globally normalised using each subject's whole brain volume. Age and gender were used as regressors of no interest in the model.

Differences were considered significant at a threshold of *p* < 0.05 corrected for multiple comparisons (family wise error correction).

Correlation of structural changes with epilepsy duration, age of onset and monthly seizure frequency at the time of scan were explored by regressing the grey matter maps against these variables.

## Results

**Figure 1:**
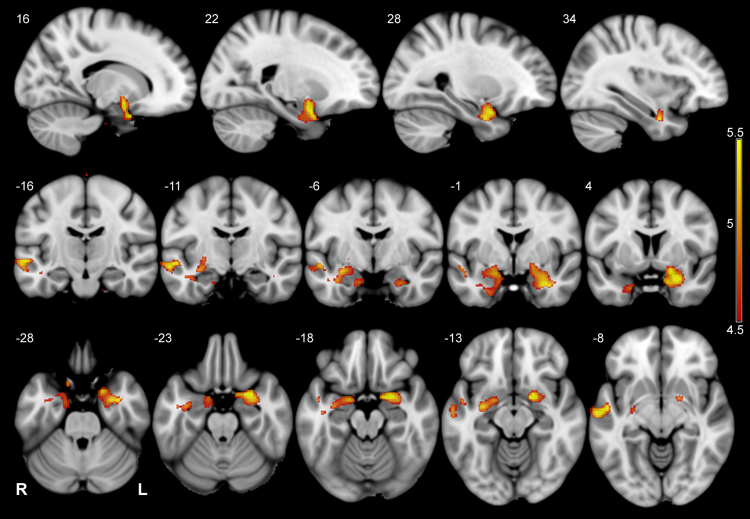
Grey matter abnormalities in patients with FLE. Greater grey matter regional volumes in FLE patients relative to controls are seen in piriform cortex and amygdala bilaterally, and on the left mid temporal lobe gyrus. Statistic maps are overlaid on an average T1 MNI template. For display proposes, maps have been thresholded at *p* < 0.005 (FDR corrected). Scale bar represent *t*-values. Numbers indicate *X*, *Z* and *Y*-coordinates in MNI space.

FLE patients showed bilaterally, predominantly right-sided increases of grey matter volumes compared to controls in the piriform cortex, amygdala and parahippocampal gyrus as well as in the left mid temporal lobe gyrus (Fig. 1). Changes in medial temporal lobes were similarly distributed in patients with left and right FLE (Supplementary Fig. 1).

Supplementary Fig. 1 related to this article can be found, in the online version, at http://dx.doi.org/10.1016/j.eplepsyres.2014.03.001.

**Supplementary Fig. I:**
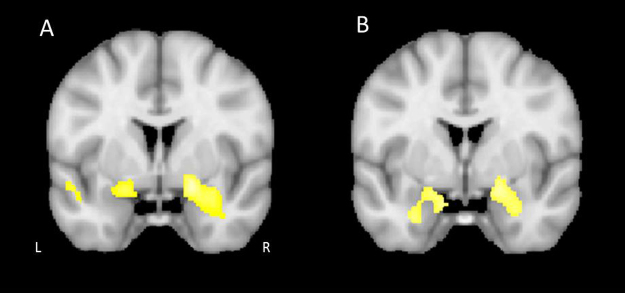
Areas of increased grey matter in (A) Left FLE and (B) Right FLE. Areas of change are bilaterally distributed with a right predominance in both subgroups of FLE.

Regression analysis did not reveal any significant correlation of GMV changes with age at seizure onset, duration of epilepsy, or seizure frequency.

There were no common areas of decreased GMV across all FLE patients, or within the left or right FLE subgroup.

## Discussion

Our study provides evidence for common cerebral structural abnormalities in patients with FLE. VBM analysis showed increased grey matter in the anterior medial temporal lobe and orbitofrontal cortex, comprising piriform cortex (temporal and frontal portion), amygdala and parahippocamapal gyrus. These findings provide further evidence for the involvement of the piriform cortex in the epileptogenic network in patients with focal epilepsies of temporal and frontal lobe origin (Laufs et al., 2011). EEG-fMRI showed that this area was commonly activated during interictal epileptic activity regardless of the location of seizure focus. Additionally, [^11^C]-flumazenil PET analysis found decreased benzodiazepine-GABAA receptor density correlated with seizure frequency in the same area. Using dynamic causal modelling, we reported recently that this structure is the driving input in an epileptogenic network supporting reading-induced foal seizures (Vaudano et al., 2012). This converging evidence from different functional imaging techniques in different focal epilepsy populations suggest that this area may have a seizure-modulating role in man, similarly to what has been observed in animals models (Piredda and Gale, 1985).

The piriform cortex and amygdalar nuclei are known to play a crucial role acting as a seizure generator in response to chemical and electrical stimulation and as an amplifier of epileptic activity when seizures are generated elsewhere. Animals studies have shown structural chronic inflammatory changes such as astrogliosis occurs in response to seizure activity in these areas (Loscher and Ebert, 1996).

Decreases and increases in GMV have been reported in different epilepsy syndromes. Volumetric measures of piriform cortex and periamygdalar cortex on autopsy specimens of TLE patients have shown atrophy in the ipsilateral side to the epileptic focus and a bilateral atrophy in up to 18% of the cases (Goncalves Pereira et al., 2005). Increases of GMV have been identified in the frontal lobes, cingulate, insula, lateral temporal lobe cortex and amygdala contralateral to the seizure focus in TLE patients (Keller et al., 2002).

In our study, VBM analysis did not detected common areas of atrophy, or decreased GMV in FLE patients. Given the high variability of seizure focus location in patients with FLE, it is not surprising that a voxel wise analysis does not identify a common area of atrophy in this population, in the way it is seen in TLE patients with hippocampal sclerosis (Bernasconi et al., 2004; Keller et al., 2002). Cortical thickness (Widjaja et al., 2011) and frontal lobe volume measures (Lawson et al., 2002) were reduced in the frontal lobes of paediatric FLE patients indicating a more widespread effect of FLE on the developing paediatric brain.

Areas of GMV decrease are generally interpreted as consequence of seizure propagation (Bernasconi et al., 2004; Keller et al., 2002), but the neuropathological correlates and biological meaning of increased volumes remote to the epileptic focus is unclear. Anatomo-pathological studies revealed the presence of mild abnormalities in the layering and cellularity of grey and white matter tissue of patients with epilepsy (Eriksson et al., 2005). These areas of microscopic dysplastic changes may offer an explanation for increased GMV detected in VBM studies (Keller et al., 2002; Woermann et al., 1999). Further studies investigating the anatomo-pathological correlates of VBM findings are needed to in order to understand the pathological role of these changes.

Our analysis did not reveal significant correlations with age at seizure onset, duration or number of seizures. The observed changes in the piriform cortex and adjacent areas are therefore, unlikely to be consequence of seizure activity but may instead represent a common node in the intrinsic epileptogenic network.

## Conclusions

Structural abnormalities shown using voxel wise analysis in patients with FLE suggest the presence for common underlying major hubs in the epileptogenic networks in focal epilepsies and add further evidence for the involvement of the piriform cortex and adjacent structures in the epileptogenic circuit of focal epilepsies.

## Figures and Tables

**Table 1:** Population demographics. Values displayed represent the mean (range) FCD: focal cortical dysplasia. AED: number of antiepileptic drugs.

	*N*	Gender (F)	Age	Age at epilepsy onset	Duration epilepsy	Aetiology	Seizures/month	AED
Controls	25	15	31 (23–55)					
Left FLE	26	10	35 (18–59)	10.6 (3–31)	24.6 (7–47)	5 FCD, 21 cryptogenic	61 (1–720)	3 (2–5)
Right FLE	17	9	31.8 (18–49)	11.3 (2–25)	19 (3–37)	6 FCD, 11 cryptogenic	138 (1–750)	3 (2–5)

## References

[bib0005] Bernasconi N., Duchesne S., Janke A., Lerch J., Collins D.L., Bernasconi A. (2004). Whole-brain voxel-based statistical analysis of gray matter and white matter in temporal lobe epilepsy. Neuroimage.

[bib0010] Eriksson S.H., Malmgren K., Nordborg C. (2005). Microdysgenesis in epilepsy. Acta Neurol. Scand..

[bib0015] Goncalves Pereira P.M., Insausti R., Artacho-Perula E., Salmenpera T., Kalviainen R., Pitkanen A. (2005). MR volumetric analysis of the piriform cortex and cortical amygdala in drug-refractory temporal lobe epilepsy. AJNR Am. J. Neuroradiol..

[bib0020] Keller S.S., Wieshmann U.C., Mackay C.E., Denby C.E., Webb J., Roberts N. (2002). Voxel based morphometry of grey matter abnormalities in patients with medically intractable temporal lobe epilepsy: effects of side of seizure onset and epilepsy duration. J. Neurol. Neurosurg. Psychiatry.

[bib0025] Laufs H., Richardson M.P., Salek-Haddadi A., Vollmar C., Duncan J.S., Gale K., Lemieux L., Loscher W., Koepp M.J. (2011). Converging PET and fMRI evidence for a common area involved in human focal epilepsies. Neurology.

[bib0030] Lawson J.A., Cook M.J., Vogrin S., Litewka L., Strong D., Bleasel A.F., Bye A.M. (2002). Clinical, EEG, and quantitative MRI differences in pediatric frontal and temporal lobe epilepsy. Neurology.

[bib0035] Loscher W., Ebert U. (1996). The role of the piriform cortex in kindling. Prog. Neurobiol..

[bib0040] Norden A.D., Blumenfeld H. (2002). The role of subcortical structures in human epilepsy. Epilepsy Behav..

[bib0045] Piredda S., Gale K. (1985). A crucial epileptogenic site in the deep prepiriform cortex. Nature.

[bib0050] Vaudano A.E., Carmichael D.W., Salek-Haddadi A., Rampp S., Stefan H., Lemieux L., Koepp M.J. (2012). Networks involved in seizure initiation. A reading epilepsy case studied with EEG-fMRI and MEG. Neurology.

[bib0055] Widjaja E., Mahmoodabadi S.Z., Snead O.C., Almehdar A., Smith M.L. (2011). Widespread cortical thinning in children with frontal lobe epilepsy. Epilepsia.

[bib0060] Woermann F.G., Free S.L., Koepp M.J., Sisodiya S.M., Duncan J.S. (1999). Abnormal cerebral structure in juvenile myoclonic epilepsy demonstrated with voxel-based analysis of MRI. Brain.

